# Prevalence of Cachexia and Outcomes in Patients With Chronic Diseases: A National Database Analysis of 5 484 103 Hospitalisations

**DOI:** 10.1002/jcsm.13688

**Published:** 2025-01-20

**Authors:** Mitja Lainscak, Tina Zupanic, Daniel Omersa, Ivan Erzen, Jerneja Farkas

**Affiliations:** ^1^ Faculty of Medicine University of Ljubljana Ljubljana Slovenia; ^2^ General Hospital Murska Sobota Murska Sobota Slovenia; ^3^ National Institute of Public Health Ljubljana Slovenia; ^4^ University Medical Center Ljubljana Ljubljana Slovenia

**Keywords:** cachexia, cancer, chronic kidney disease, chronic obstructive pulmonary disease, heart failure, mortality

## Abstract

**Background:**

Cachexia is a frequent companion of chronic diseases and a well‐established predictor of poor patient performance and outcome. Since cachexia as a discharge diagnosis is not much investigated, we aimed to investigate prevalence of cachexia in hospitalised patients and their outcome.

**Methods:**

We conducted a retrospective analysis of the National Hospital Health Care Statistics Database using the 10th revision of the International Classification of Diseases codes. During period 2004–2019, patients with cachexia were identified, as well as patients with cancer, heart failure, chronic obstructive pulmonary disease and chronic kidney disease. The primary endpoint was the discharge code of cachexia; secondary endpoints were length of hospital stay, in‐hospital and post discharge all‐cause mortality.

**Results:**

In period 2004–2019, 5 484 103 hospitalisations were screened; cachexia was coded 19 348 times (0.35%) in 14 089 patients (67 ± 13 years, 42% women). From 2004 to 2019, prevalence of cachexia increased steadily from 1.2% to 1.9%, which was most prominent for cancer and chronic obstructive pulmonary disease. At one year post discharge, 49% patients with cachexia were dead as compared to 26% in patients without cachexia. In Cox multivariate analysis, cachexia predicted post‐discharge death in any of chronic diseases (hazard ratio of 1.28 in heart failure to 1.47 in chronic kidney disease).

**Conclusions:**

In our report from a National Hospital Health Care Statistics Database, we found that cachexia was underreported as ICD‐10 coded discharge diagnosis in patients with chronic diseases. When diagnosed, it was associated with higher hazard of post discharge mortality.

## 1 Introduction

Cachexia is a frequent companion of chronic diseases and a well‐established predictor of poor patient performance and outcome [1]. Along chronic disease trajectory, cachexia usually ensues as a final and irreversible phase. Several cachexia definitions have developed over time; while some of which are disease specific, the hallmark criteria of disease‐related body wasting with reductions in patient performance and well‐being is uniform [2, 3]. Even though measures to treat and prevent cachexia were intensively investigated, our approach remains largely symptomatic, with few promising strategies in sight [4, 5, 6, 7, 8]. Similarly, epidemiological data is scarce, lacks uniform definition, rarely extends beyond a single or few centres and is mostly limited to a single chronic disease [9, 10, 11, 12]. In view of patients related outcomes in terms of quality of life and mortality but also regarding the healthcare system at large through hospitalisations and associated costs, better insight into epidemiology is necessary.

Irrespective of definition used [2, 3], despite differences in diagnostic and prognostic performance, a physician who evaluates a patient with chronic disease has a central role to consider cachexia and diagnose it if diagnostic criteria are met. Hospitalisations are frequent in patients with chronic diseases [13], which open a window of opportunity for comprehensive patient assessment beyond main condition which can be relevant for risk stratification and management [14]. Diagnosing cachexia should not be overly time consuming, particularly when knowing that up to 75% of hospitalised patients have reduced food intake [15] and that routine in‐hospital laboratory testing includes at least some biochemical markers required for diagnosis [2], which in cancer even is not necessary [3]. When cachexia is diagnosed, it should be coded as a discharge diagnosis. It is therefore surprising that cachexia as a discharge diagnosis is not much investigated [16].

Since most countries worldwide do not have the capacity and appropriate central registries, national sample analyses are generally infrequent. Overall, hospitalisations, mortality, and drug prescription are best reported in the literature and Slovenia is among those countries that already contributed [17]. Current analysis explored the National Hospital Health Care Statistics Database, which captures hospital admissions and discharges and is linked it with Central population registry to capture mortality data. We aimed to investigate prevalence of cachexia as a discharge diagnosis in all patients and in patients with chronic diseases (cancer, heart failure, chronic obstructive pulmonary disease ‐ COPD and chronic kidney disease), and post discharge mortality.

## 2 Methods

### 2.1 Study Design and Patients

We conducted a retrospective analysis of the National Hospital Health Care Statistics Database of the National Institute of Public Health that captures nationwide hospitalisation data. Every hospital in Slovenia is required by law to report a unique data set for every hospitalisation to the database using a standardised methodology and the 10th revision of the International Classification of Diseases (ICD‐10).

**FIGURE 1:**
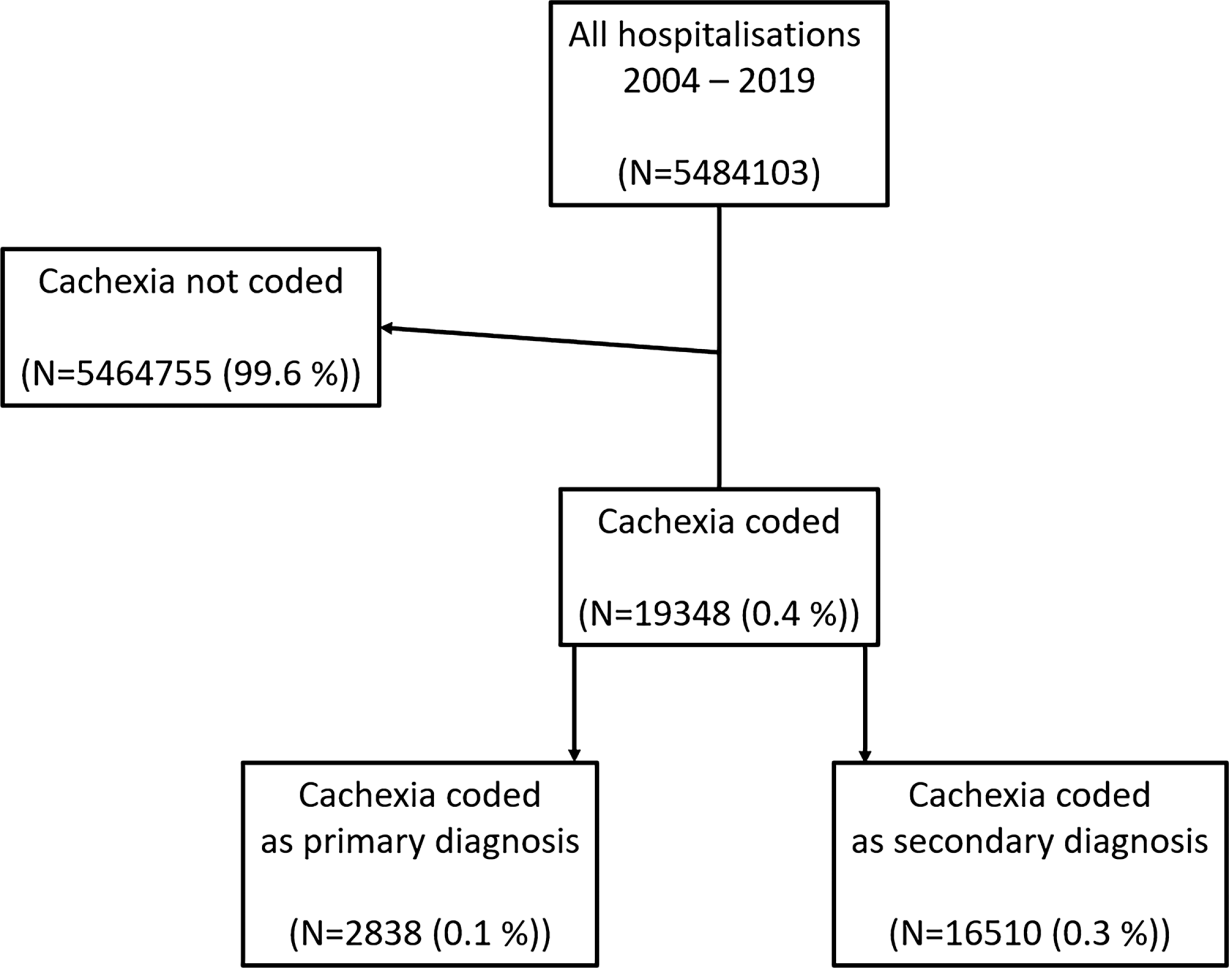
Cachexia as discharge diagnosis in Slovenian hospitals in the period 2004–2019.

For the period 2004–2019, the database was searched for cachexia as a primary or any of the discharge diagnoses, using the ICD‐10 codes R64, C80.9 and B22.2 coded as the main (principal) or any other diagnosis. Patients aged 18 or more and discharged with any of the codes in the selected period were identified. From the database, the unique patient identifier, hospital stay, sex, age, discharge diagnoses, region of permanent residence and date of death were documented for every patient. We excluded all patients with permanent residence outside Slovenia, and those with a mismatch between date of death and date of admission (Figure 1). Specific co‐morbidity was defined as a discharge diagnosis in accordance with individual ICD‐10 codes as follows: cancer (C00–C96), heart failure (I50.0–I50.9, I42.0–I42.9, I11.0, I13.0, I13.2 and J81), COPD (J43.2, J43.8, J43.9, and J44.0‐J44.9), and chronic kidney disease (N18.0–N18.9). This study was conducted in accordance with the Declaration of Helsinki and the protocol was approved by the National Medical Ethics Committee (approval no. 0120‐475/2019/7).

### 2.2 Study Endpoints and Statistical Analysis

The primary endpoint of the study was the discharge code of cachexia; secondary endpoints were length of hospital stay, in‐hospital and post discharge all‐cause mortality. Data were analysed with the SPSS 25 program. Chi‐square test was used to compare groups with primary and secondary cachexia diagnosis by sex. One‐way ANOVA analysis of variance was used to compare mean and median age, length of stay and comorbidities. Cox regression models were built to determine the association of potential risk factors with mortality with selected chronic disease as dependant variables and sex, age and cachexia as independent variables. Hazard ratios (HR) with 95% confidence intervals (95% CI) were calculated for each model. Survival curves were plotted using Kaplan–Meier method to compare the survival probability at different points of time and to compare among patients with selected chronic diseases between the group with cachexia and the group without it. The value of *p* ≤ 0.05 was considered as significant.

## 3 Results

**FIGURE 2:**
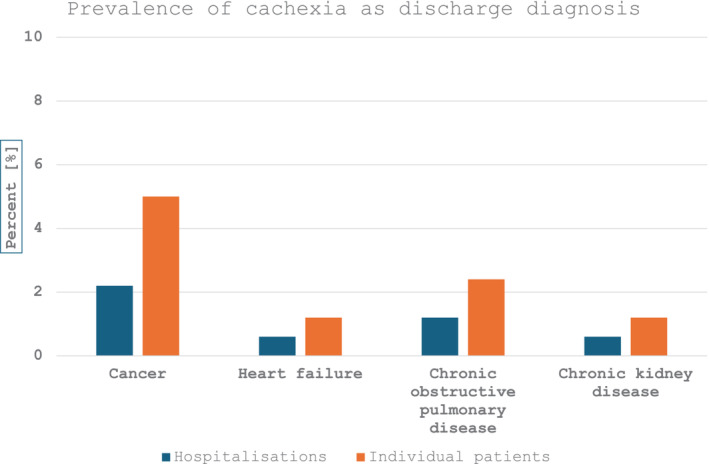
Prevalence of cachexia at discharge or death in hospitalisations and individual patients.

**FIGURE 3:**
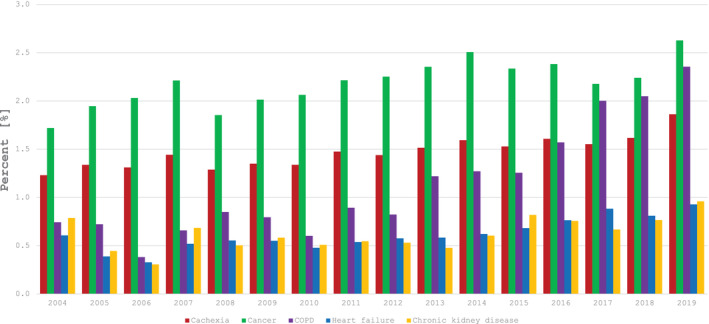
Prevalence of cachexia at discharge or death in the period 2004–2019 in patients with cancer, chronic obstructive pulmonary disease, heart failure and chronic kidney disease.

In period 2004–2019, registry recorded 5 484 103 hospitalisations and cachexia was coded 19 348 times (0.35%), mostly as secondary diagnosis (*N* = 16 510, 85.3%)—Figure 1 and Table S1. Cachexia was reported in 10 835 individual patients (in 1814 or 16.7% as primary diagnosis), who were hospitalised 14 889 times; their mean age was 67 ± 13 years and 42% were women. Most hospitalisations and individual patients had concomitant diagnosis of cancer (*N* = 12 649 or 65.4% and *N* = 9085 or 64.5%), followed by heart failure, COPD and chronic kidney disease; these chronic diseases were diagnosed at discharge in 995 192 hospitalisations in 312 190 individual patients. Patients with cachexia had longer in hospital stay and had more comorbidities than those without cachexia—Table 1. When cachexia was the primary discharge diagnosis, patient length of stay was shorter, they had less comorbidity and generally had lower in‐hospital mortality than those with cachexia as secondary diagnosis. Figure 2 demonstrates prevalence of cachexia diagnosis in different chronic diseases, which ranged from 0.6% in chronic kidney disease and heart failure to 2.2% in cancer. The prevalence doubled if analysed per individual patient: from 1.2% in heart failure to 5% in cancer. From 2004 to 2019, prevalence of cachexia increased steadily from 1.2% to 1.9%, which was most prominent for cancer and COPD—Figure 3.

**TABLE 1:** Baseline characteristics.

		Cachexia		No cachexia
	Primary diagnosis	Secondary diagnosis	Total	Total
All				
Hospitalisations	2838	16 510	19 348	995 192
Individual patients	2173	11 916	14 089	310 972
Age (years)	66 ± 13	67 ± 15	67 ± 15	68 ± 16
Men	54%	57%	57%	53%
Length of stay (days)	11 ± 12	15 ± 22	14 ± 21	10 ± 22
In‐hospital death	7.9%	11.4%	10.8%	8.6%
Comorbidities	3 ± 2	5 ± 3	5 ± 3	4 ± 3
Cancer				
Hospitalisations	2372	10 277	12 649	567 054
Individual patients	1751	7334	9085	181 124
Age (years)	66 ± 12	66 ± 13	66 ± 13	64 ± 16
Men	54%	59%	58%	54%
Length of stay (days)	10 ± 12	13 ± 16	13 ± 15	9 ± 26
In‐hospital death	9.4%	11.0%	10.7%	5.6%
Comorbidities	2 ± 2	4 ± 3	3 ± 3	3 ± 2
Heart failure				
Hospitalisations	147	1748	1895	299 837
Individual patients	139	1561	1700	136 900
Age (years)	75 ± 12	76 ± 13	76 ± 13	76 ± 12
Men	50%	53%	53%	48%
Length of stay (days)	13 ± 12	16 ± 20	16 ± 20	12 ± 15
In‐hospital death	37.6%	32.1%	32.7%	18.4%
Comorbidities	4 ± 3	7 ± 4	7 ± 4	5 ± 3
Chronic obstructive pulmonary disease				
Hospitalisations	62	1274	1336	114 074
Individual patients	55	951	1006	42 437
Age (years)	70 ± 9	70 ± 10	70 ± 10	71 ± 12
Men	74%	67%	67%	70%
Length of stay (days)	12 ± 10	16 ± 19	16 ± 19	11 ± 14
In‐hospital death	3.9%	8.8%	8.4%	7.1%
Comorbidities	5 ± 2	5 ± 4	5 ± 4	5 ± 3
Chronic kidney disease				
Hospitalisations	61	930	991	155 796
Individual patients	59	773	832	63 817
Age (years)	75 ± 12	72 ± 16	72 ± 16	73 ± 14
Men	66%	63%	63%	56%
Length of stay (days)	14 ± 11	17 ± 22	17 ± 22	11 ± 14
In‐hospital death	24.2%	43.1%	41.7%	11.0%
Comorbidities	5 ± 3	8 ± 4	7 ± 4	6 ± 3

*Note:* Data are presented as number or proportion or as mean ± standard deviation.

**FIGURE 4:**
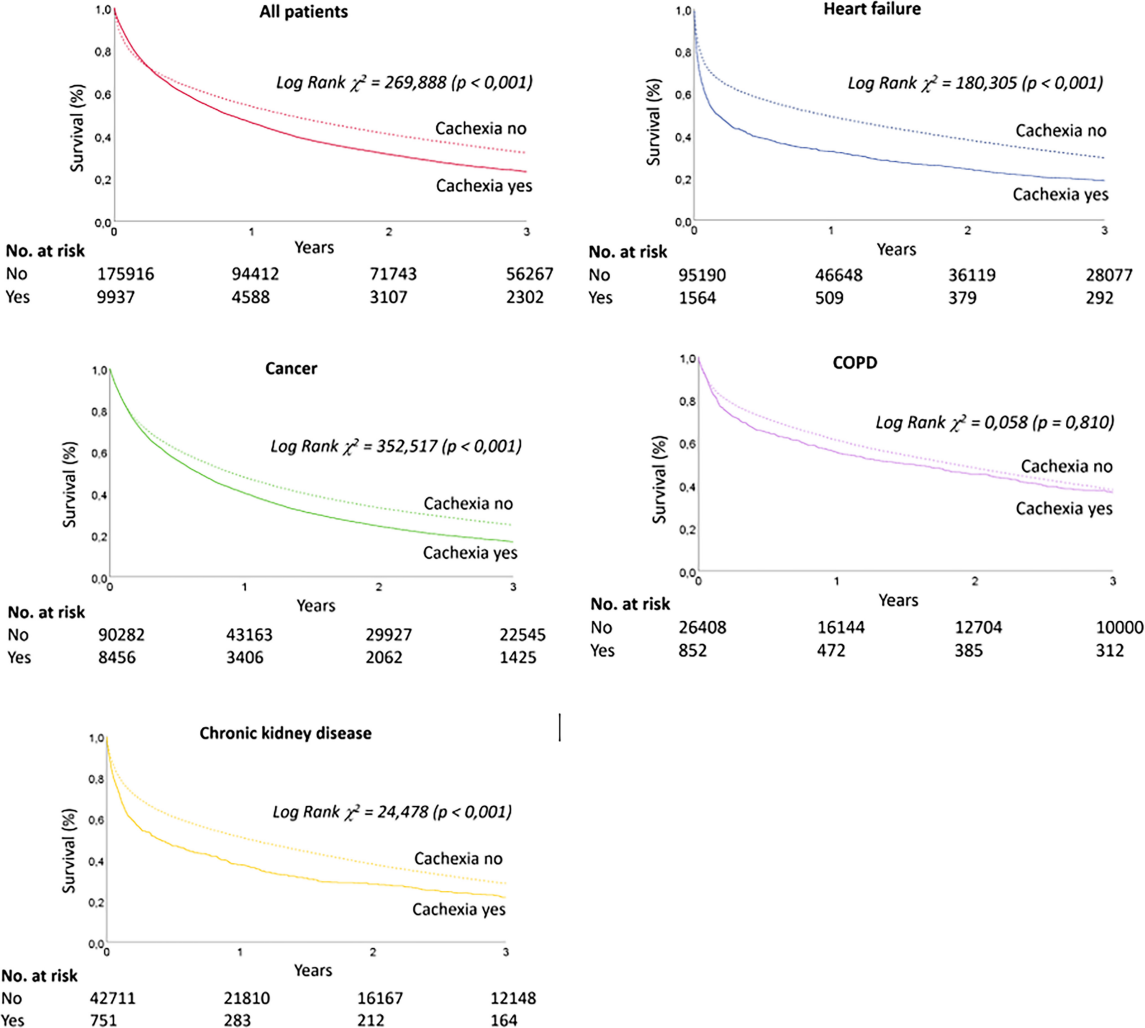
Survival of patients with or without cachexia: a) all patients, b) cancer, c) chronic obstructive pulmonary disease, d) heart failure and e) chronic kidney disease.

**FIGURE 5:**
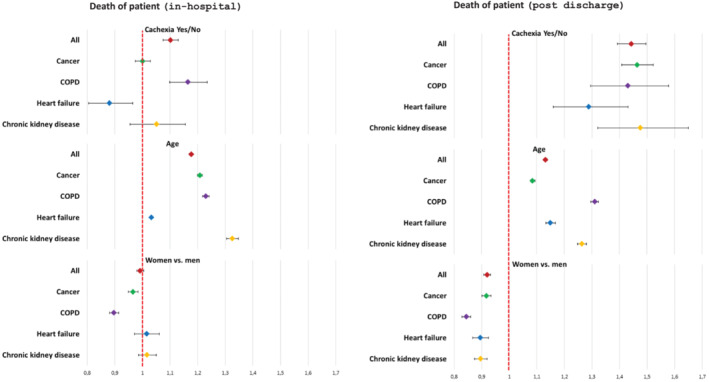
Cox models of proportional hazards for a) in‐hospital and b) post‐discharge mortality for patients with/without cachexia, per one‐year increase in age and in women vs. men.

During index hospitalisation, cachexia was associated with about two times higher mortality (CKD 3.7 times, about 1.7 times for other diseases) that extended into post discharge period in all chronic diseases except for COPD (Table 1 and Figure 4). At one year post discharge, 49% patients with cachexia were dead as compared to 26% in patients without cachexia (Figure 4). In Cox multivariate analysis (Figure 5), age was associated with a higher hazard of in‐hospital death, while cachexia was significant only in patients with heart failure and COPD (Figure 5a). In patients discharged alive, cachexia was associated with 30–50% increased hazard of death, as was male sex and age (Figure 5b).

## 4 Discussion

To best of our knowledge, our report is a first long‐term national hospitalisation database analysis of cachexia coded using ICD‐10 at discharge or death. Our most relevant findings are: i) cachexia prevalence in chronic diseases if estimated using discharge diagnosis is lower than previously estimated; ii) cachexia is most frequently reported in patients with cancer; iii) cachexia is associated with longer in‐hospital stay and higher hazard of post discharge mortality.

Cachexia meets the criteria for public health problem [18], which should prompt clinicians to screen and diagnose cachexia when seen in general practice, outpatient clinics and even more so in hospitalised patients. This, however, is not the case as evident from few studies, which used ICD‐10 cachexia code and large hospitalisation registries to investigate cachexia diagnosis in patients with cancer [16, 19]. Once hospitalised, patients with chronic diseases can be evaluated with regard to their main disease and beyond. The latter should ideally include assessment of nutritional status and body wasting, including cachexia. Conveniently, the nutritional screening is possible as evident from the Nutrition day survey [15]; yet the implementation in clinical practice is far from satisfactory although poor nutritional status is associated with lower quality of life and worse outcome [20]. A lack for cachexia screening and reporting may be manifold, starting from inability to apply diagnostic criteria due to costs or insufficient human resources but also due to lack of robust strategies to improve patient outcome [8]. Our data demonstrate that cachexia is reported as discharge diagnosis far less frequently than it is reported or estimated in recent literature [9, 10, 11, 12]. Our analysis was not designed to investigate the cause for lower prevalence and we also were not able to determine which criteria for cachexia diagnosis, if any, were applied. What is encouraging is the fact that an increase of cachexia coded at discharge over the years was observed. Again, we are unable to say whether this was due to higher awareness about cachexia, relevant literature about cachexia diagnosis published during the study period [2, 3] or maybe due to better therapies for underlying chronic disease that prevented death early in the natural course with more patients surviving into advanced stages, when cachexia is more frequent. In any case, it is important to recognise whether cachexia is present as this can modify patient management towards quality of life measures and potentially also towards end of life management [8].

In our study, cachexia was diagnosed mostly in patients with cancer, which may reflect general understanding among health care professionals and patients: that cancer is one of most severe chronic diseases that eventually will lead to body wasting and cachexia. Globally, in terms of morbidity and mortality, cardiovascular diseases remain the most relevant [13] and heart failure mortality is at least comparable if not worse than in cancer in general [21]. This should alert health care professionals and patients about outcome in non‐malignant chronic disease and the need to implement guideline directed management that can delay or prevent cachexia [22]. When comparing the patients with individual chronic disease, those with cancer were younger, less frequently men and were hospitalised fewer days less than those with heart failure, COPD and chronic kidney disease. The latter could reflect shorter hospitalisations related to targeted interventions that are less established for patients other than cancer. In any case, efforts towards diagnosing body wasting and cachexia should be shifted to early stages in disease trajectory when more interventions are available with potential to improve quality of life and patient well‐being [3].

All‐cause mortality, both in‐hospital and post discharge, was high and expectedly related to age. Co‐existing cachexia also increased hazard of post‐discharge mortality for all patients, while during hospitalisation, the risk was higher only for patients with COPD whereas the risk was lower in patients with heart failure. Exact reasons are beyond the capacity of this study but it is plausible that clinicians were less likely to code cachexia if patient died in hospital. Taken together, it is even more important to screen for cachexia during hospitalisation as some of the suggested criteria like laboratory markers are routinely assessed when in hospital, and questions related to weight loss, fatigue and anorexia should be part of clinical examination on admission or during hospital stay. When comparing the mortality post discharge, about 60% of patients with cachexia died within 12 months. This amplifies our previous statement about general measures to improve quality of life and end of life management in particular [23].

Our analysis confirms discordance between estimates of cachexia prevalence and what is reported in clinical practice [1, 9, 10, 11, 12, 16, 19]. Cachexia always was related to very advanced disease stage although patients can meet diagnostic criteria (much) prior to advanced stages of the disease. Awareness about the presence and importance of body wasting is likely low among clinicians [24], potentially also due to the fact there is no global activity or awareness day assigned specifically to body wasting. In other diseases, very active public awareness campaigns focusing on a single day or period annually, in many cases with recognisable ribbon (e.g. pink for breast cancer) contribute significantly to greater recognition and understanding among lay public, patients, health care professionals and policy makers. Next step should be to promote regular body wasting evaluation when patients come in contact with health care professionals, particularly during hospitalisation. For this purpose, a stepwise approach like for frailty [25] rather than all‐or‐nothing approach could be considered.

We acknowledge limitations of our analysis. Per methodology, only cachexia coded as discharge diagnosis was captured which does not mean it was not diagnosed and mentioned in the patient medical charts; such discrepancy was already reported [26] and cannot be excluded in our analysis. Chronic disease was defined with ICD‐10 code, which may not be completely accurate as we do not have insight whether diagnostic criteria for individual chronic disease were applied as appropriate and whether all diagnoses were always coded. These limitations, however, are methodology driven and shared with other studies alike.

In conclusion, cachexia seems to be underreported as ICD‐10 coded discharge diagnosis in patients with chronic diseases. If reported proportionate between chronic diseases, then prevalence in cancer was about twice higher than in other chronic diseases. In patients discharged alive, cachexia increased hazard of death for 30–50% thus hospitalisation should be perceived as window of opportunity to screen for cachexia and to consider chronic disease targeted interventions and other interventions to improve quality of life.

## Conflicts of Interest

Mitja Lainscak reports receiving grants from Slovenian Research Agency, personal fees or honoraria from Boehringer Ingelheim, AstraZeneca, and Novartis.

Tina Zupanic reports no disclosures.

Daniel Omersa reports no disclosures.

Ivan Erzen reports receiving grants from Slovenian Research Agency.

Jerneja Farkas reports receiving grants from Slovenian Research Agency.

## Supporting information


**Table S1** Cachexia codes in patients with chronic disease.
