# Supplementary material for: Prevalence of Cachexia and Outcomes in Patients With Chronic Diseases: A National Database Analysis of 5 484 103 Hospitalisations
Source: J Cachexia Sarcopenia Muscle. 2025 Jan 20;16(1):e13688. doi: 10.1002/jcsm.13688 (PMC11744301; doi:10.1002/jcsm.13688)
Supplement: Supplementary file 1 — Table S1 Cachexia codes in patients with chronic disease. [file JCSM-16-e13688-s001.docx]

Supplementary Table 1. Cachexia codes in patients with chronic disease.

| International Classification of Diseases (ICD-10) code | Hospitalisations |
| --- | --- |
| R64 - Cachexia | 12785 (66.1%) |
| C809 – Malignant neoplasm, primary site unspecified, malignant cachexia | 6372 (32.9%) |
| B222 – HIV disease resulting in wasting syndrome | 41 (0.2%) |
| R64 and C809 | 150 (0.8%) |
